# Association of circulating branched-chain amino acids with risk of moyamoya disease

**DOI:** 10.3389/fnut.2022.994286

**Published:** 2022-09-02

**Authors:** Chaofan Zeng, Peicong Ge, Chenglong Liu, Xiaofan Yu, Yuanren Zhai, Wei Liu, Qiheng He, Junsheng Li, Xingju Liu, Jia Wang, Xun Ye, Qian Zhang, Rong Wang, Yan Zhang, Jizong Zhao, Dong Zhang

**Affiliations:** ^1^Department of Neurosurgery, Beijing Tiantan Hospital, Capital Medical University, Beijing, China; ^2^China National Clinical Research Center for Neurological Diseases, Beijing, China; ^3^Center of Stroke, Beijing Institute for Brain Disorders, Beijing, China; ^4^Beijing Key Laboratory of Translational Medicine for Cerebrovascular Disease, Beijing, China; ^5^Beijing Translational Engineering Center for 3D Printer in Clinical Neuroscience, Beijing, China; ^6^Department of Neurosurgery, Beijing Hospital, Beijing, China

**Keywords:** moyamoya disease, branched-chain amino acids (BCAAs), metabolites, biomarkers, risk factors

## Abstract

**Objective:**

Branched-Chain Amino Acids (BCAAs) has been identified as a risk factor for circulatory disease. Nevertheless, the effects and mechanisms of BCAAs on the risk of moyamoya disease (MMD) remain unrecognized. Hence, we aimed to elucidate the association between circulating BCAAs and the risk of MMD and clinical subtypes.

**Methods:**

We conducted a case-control study of 360 adult MMD patients and 89 matched healthy controls consecutively recruited between September 2020 and December 2021. Serum level of BCAAs was quantified by liquid chromatography-mass spectrometry. The associations between BCAAs and risk of MMD were evaluated.

**Results:**

Increased level of serum BCAAs was observed in MMD patients (*P* < 0.001). After adjusting for traditional confounders, the elevated BCAAs level was significantly associated with the risk of MMD (Q4 vs. Q1: odds ratio, 3.10 [95% CI, 1.29–7.50]). The risk of subtypes in MMD also increased with each increment in the quartiles of BCAAs. Furthermore, BCAAs offered substantial improvement in risk reclassification and discrimination for MMD and subtypes.

**Conclusion:**

Higher level of circulating BCAAs was associated with increased risk of MMD and clinical subtypes. This study will help to elucidate the pathogenesis of MMD, which may provide the support for facilitating the treatments and preventions.

## Introduction

Moyamoya disease (MMD), characterized by progressive stenosis of distal portion of internal carotid arteries and abnormal collaterals at the base of brain, is recognized as the main cause of stroke in East Asians (1). MMD has been considered as a multifactorial disease, caused by genetic, immune, inflammation and other factors (2). Although the *RNF213* variants have been identified to be associated with angiopathy in MMD, the frequency of variants was quite low in China (2–4). Our recent study has demonstrated that traditional modifiable factors were related to the risk of MMD (5), while the well-known risk factors cannot fully account for the etiology of MMD.

Recently, progress in high-throughput multi-omics technologies has provided new insight into the pathogenesis of diseases (6). Circulating metabolites reveal the information of systemic alterations and disease mechanisms. and could act as biomarkers that accurately estimate the risk of stroke (6). Branched-Chain Amino Acids (BCAAs), consisting of leucine, isoleucine, and valine, is a compound of essential amino acids that regulates diverse functions, including cell growth, autophagy, and lipid metabolism (7). BCAAs mainly participates in biological activities by activating the mammalian target of rapamycin (mTOR) pathway. It has been shown to be associated with metabolic disorders, cardiovascular diseases and cancer (8–10). Despite few metabolomics studies have been performed in MMD patients (11, 12), the targeted outcomes and potential mechanisms of BCAAs in MMD was hitherto unrecognized.

In the current study, we enrolled a large population of MMD patients and healthy controls (HCs) and analyzed the characteristics of circulating BCAAs in MMD. We aimed to demonstrate the association of the serum BCAAs level with the risk of MMD and clinical subtypes. This work will help to identify novel biomarkers, and elucidate the pathogenesis of MMD, which may provide the support for improving the interventions and preventions.

## Materials and methods

### Study design and participants

**Figure 1:**
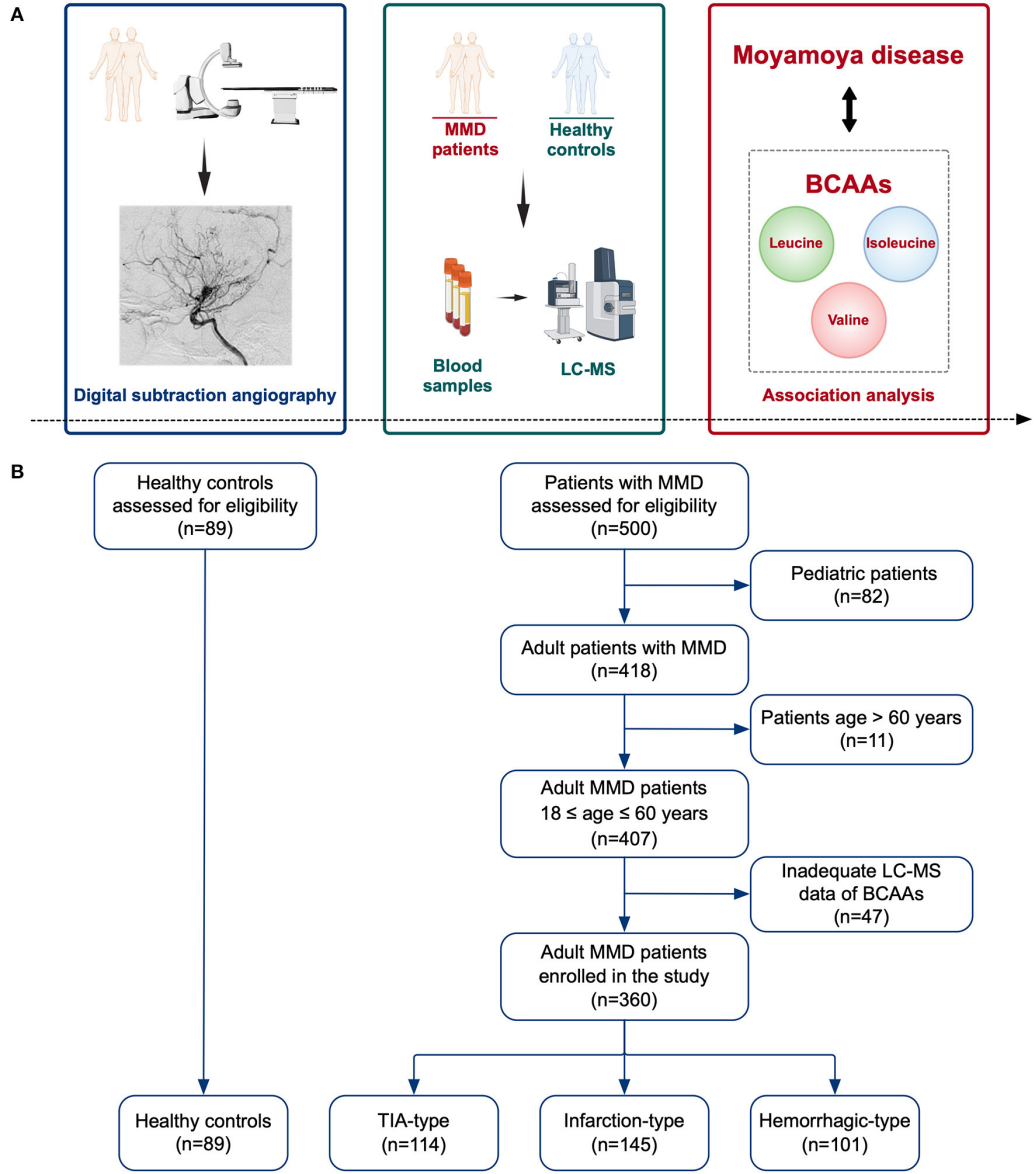
Schematic diagram of the study. **(A)** Illustration of the study methods and purpose. **(B)** Flow chart of the study participants. MMD, moyamoya disease; LC-MS, liquid chromatography-mass spectrometry; BCAAs, branched-chain amino acids; TIA, transient ischemic attack.

In this study, we prospectively recruited adult MMD patients at the Department of Neurosurgery, Beijing Tiantan Hospital from September 2020 to December 2021. Eligible patients were age 18–60 years, unilateral and bilateral MMD diagnosed by digital subtraction angiography (DSA) following the Japanese guidelines (1). Patients were excluded if they refused to participate in the study or had inadequate Liquid chromatography-mass spectrometry (LC-MS) data of BCAAs. Finally, 360 adult patients with complete measurement of BCAAs were enrolled in the study, consisting of 114 patients of transient ischemic attack (TIA)-type MMD, 145 patients of infarction-type MMD, and 101 patients of hemorrhagic-type MMD (Figure 1). Besides, 89 age-matched HCs who underwent routine physical examination were recruited. The HCs generally had no comorbidities. The study was approved by the Ethics Committee of Beijing Tiantan Hospital. Informed consents were obtained from all participants.

### Baseline data collection and laboratory assessment

Demographic data (age and sex), history of risk factors (hypertension, diabetes mellitus, hyperlipidemia, cigarette smoking, and alcohol drinking), clinical features (heart rate, blood pressure, body mass index [BMI]), clinical manifestations (TIA, infarction, and hemorrhage) were collected *via* chart views.

Fasting blood samples were collected after admission from all participants. Routine and biochemical blood tests were conducted to measure the levels of potential circulating biomarkers: white blood cell (WBC) count, lymphocyte (LY) count, neutrophil count, monocyte count, red blood cell (RBC) count, hemoglobin (HGB), hematocrit (HCT), mean corpuscular volume (MCV), mean corpuscular hemoglobin (MCH), mean corpuscular hemoglobin concentration (MCHC), platelet (PLT) count, glucose, creatinine, uric acid, triglyceride (TG), total cholesterol (TC), high-density lipoprotein cholesterol (HDL-C), low-density lipoprotein cholesterol (LDL-C), apolipoprotein A_1_ (ApoA_1_), apolipoprotein B (ApoB), and homocysteine (Hcy). Hcy ≥ 15.0 μmol/L was considered as hyperhomocysteinemia (HHcy). Besides, peripheral inflammatory biomarkers including neutrophil-to-lymphocyte ratio (NLR), monocyte-to-lymphocyte ratio (MLR), platelet-to-lymphocyte ratio (PLR), systemic immune-inflammation index (SII) (PLT count × neutrophil count/LY count), and monocyte-to-HDL cholesterol ratio (MHR) were calculated. Serum samples were also collected at baseline from all individuals. The serum samples were stored at −80 °C in the Central Laboratory of Beijing Tiantan Hospital. We used LC-MS techniques to quantitatively profile the serum metabolites of BCAAs. The level of BCAAs was calculated as the sum of levels of leucine, isoleucine, and valine.

### Statistical analysis

All statistics analyses were performed using SPSS version 26.0 (IBM Corporation, Armonk, NY, USA) and R version 4.1.2 (R Development Core Team). Baseline characteristics were presented and compared between MMD patients and HCs. The categorical variables were presented as frequencies, and continuous variables were expressed as mean with standard deviation (SD) or median with interquartile range (IQR). Categorical data were compared using the χ^2^ test or Fisher exact test between groups, and continuous data were compared with two-tailed Student *t*-tests or Mann-Whitney *U* tests. One-way ANOVA or Kruskal-Wallis test was used to test the trend for continuous variables across BCAAs, and the Cochran-Armitage trend χ^2^ test was conducted for categorical variables. The logistic regression models were performed to identify the independent factors for MMD and its subtypes. The crude model was the unadjusted regression model of BCAAs. The model 1 adjusted for covariates including age and sex. The model 2 further adjusted for BMI, WBC count, neutrophil count, glucose, TG, TC, HDL-C, LDL-C, APO-A_1_, Hcy, NLR, SII, and MHR. Furthermore, we evaluated the predictive performance of models for the risk of MMD and its subtypes by establishing receiver-operating characteristic (ROC) curves and calculated the area under the curve (AUC). Moreover, the performance of BCAAs in the basic model built based on traditional risk factors were assessed. The net reclassification index (NRI) and integrated discrimination improvement (IDI) were calculated in risk classification by adding BCAAs to the basic model. *P* < 0.05 was considered statistical significance.

## Results

A total of 360 MMD patients (114 cases with TIA, 145 cases with infarction, and 101 cases with hemorrhage) and 89 matched HCs were included in the study.

### Baseline characteristics and BCAAs of MMD patients and HCs

**Figure 2:**
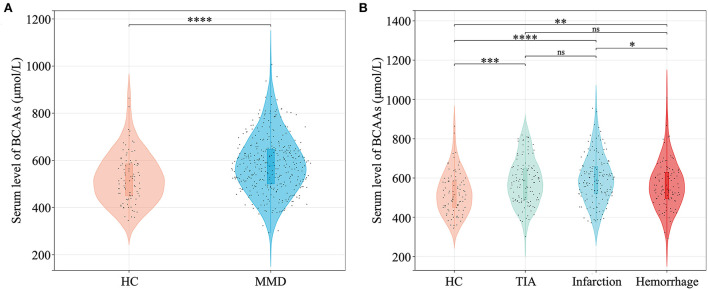
Quantitative analysis of serum BCAAs level between MMD patients and HCs. **(A)** Comparison of BCAAs level between HCs and MMD patients. **(B)** Comparison of BCAAs level between HCs and MMD subtypes. BCAAs, branched-chain amino acids; HC, healthy control; MMD, moyamoya disease; TIA, transient ischemic attack; ns, not significant. *P < 0.05; **P < 0.01; ***P < 0.001; ****P < 0.0001.

Baseline characteristics of MMD cases and HCs were shown in Table 1. History of risk factors for stroke (hypertension, diabetes mellitus, hyperlipidemia, cigarette smoking, and alcohol drinking) were more prevalent in MMD patients (*P* < 0.05 for all). In MMD patients, the levels of systolic blood pressure (SBP), diastolic blood pressure (DBP), and BMI were significantly higher than in HCs. Patients in groups of MMD subtypes had a higher level of WBC count, neutrophil count, glucose, TG, Hcy, NLR, SII, and MHR than in HC group (*P* < 0.05 for all). Levels of laboratory results including LY count, HGB, HCT, MCHC, TC, HDL-C, LDL-C, ApoA_1_, and PLR were significantly different between groups (*P* < 0.05 for all). In addition, patients with MMD and its subtypes had a significantly higher level of BCAAs than that of HCs (*P* < 0.05 for all), while patients with hemorrhagic-type MMD had a lower level of BCAAs than that of infarction-type (*P* < 0.05) (Figure 2). The significant differences of individual BCAAs (leucine, isoleucine, and valine) between MMD patients and HCs were similar to the total BCAAs (Supplementary Figure S1).

**Table 1:** Baseline characteristics of HCs and MMD patients.

**Variables**	**HCs**	**TIA**	**Infarction**	**Hemorrhage**	***P* value**
	**(*n =* 89)**	**(*n =* 114)**	**(*n =* 145)**	**(*n =* 101)**	
Age, y, mean ± SD	39.81 ± 11.57	40.51 ± 10.27	42.33 ± 9.96	41.78 ± 10.61	0.260
Sex, Female/Male	1.41:1	1.43:1	1.10:1	1.97:1	0.190
History of risk factors, n (%)
Hypertension	0 (0)	35 (30.7)	67 (46.2)	29 (28.7)	<0.001^*^
Diabetes mellitus	0 (0)	16 (14.0)	39 (26.9)	4 (4.0)	<0.001^*^
Hyperlipidemia	0 (0)	17 (14.9)	28 (19.3)	9 (8.9)	<0.001^*^
Cigarette smoking	2 (2.2)	20 (17.5)	34 (23.4)	17 (16.8)	<0.001^*^
Alcohol drinking	0 (0)	14 (12.3)	20 (13.8)	8 (7.9)	0.003^*^
Clinical features, mean ± SD
Heart rate, bpm	77.79 ± 9.73	78.77 ± 6.41	77.83 ± 6.72	79.30 ± 5.92	0.332
SBP, mmHg	123.64 ± 11.77	132.81 ± 12.09	134.16 ± 13.40	129.16 ± 12.25	<0.001^*^
DBP, mmHg	78.46 ± 8.35	81.55 ± 9.04	83.16 ± 9.68	80.32 ± 8.96	0.001^*^
BMI, kg/m^2^	23.96 ± 3.39	25.87 ± 4.88	25.96 ± 4.37	24.32 ± 4.11	<0.001^*^
Laboratory results, median ± IQR
WBC count, 10^9^/L	6.03 ± 1.88	6.93 ± 2.57	7.02 ± 2.39	6.43 ± 2.46	<0.001^*^
LY count, 10^9^/L	1.91 ± 0.71	2.08 ± 0.79	1.97 ± 0.80	1.72 ± 0.84	<0.001^*^
Neutrophil count, 10^9^/L	3.44 ± 1.62	4.24 ± 1.98	4.35 ± 1.73	3.88 ± 1.86	<0.001^*^
Monocyte count, 10^9^/L	0.35 ± 0.14	0.36 ± 0.18	0.36 ± 0.16	0.34 ± 0.17	0.289
RBC count, 10^12^/L	4.69 ± 0.65	4.64 ± 0.72	4.68 ± 0.71	4.60 ± 0.59	0.306
HGB, g/L	144.00 ± 19.00	141.50 ± 22.00	143.00 ± 27.00	137.00 ± 22.00	0.042^*^
HCT, L/L	0.42 ± 0.05	0.41 ± 0.06	0.41 ± 0.08	0.41 ± 0.05	0.041^*^
MCV, fL	90.10 ± 5.10	90.10 ± 5.90	89.20 ± 5.30	90.00 ± 5.40	0.759
MCH, pg	30.70 ± 2.00	30.95 ± 2.00	30.70 ± 2.50	30.80 ± 2.30	0.648
MCHC, g/L	341.00 ± 15.00	342.50 ± 12.00	344.00 ± 13.00	339.00 ± 12.00	0.039^*^
PLT count, 10^9^/L	233.00 ± 87.00	249.50 ± 72.00	250.00 ± 80.00	244.00 ± 76.00	0.304
Fasting glucose, mmol/L	5.04 ± 0.62	5.12 ± 1.01	5.22 ± 1.42	4.91 ± 0.67	<0.001^*^
Creatinine, μmol/L	57.70 ± 19.20	53.95 ± 20.05	57.80 ± 20.80	53.10 ± 21.75	0.271
Uric acid, μmol/L	310.60 ± 103.50	313.25 ± 119.60	312.00 ± 118.30	292.90 ± 113.30	0.135
TG, mmol/L	0.87 ± 0.62	1.24 ± 0.91	1.20 ± 0.75	1.13 ± 0.85	<0.001^*^
TC, mmol/L	4.62 ± 0.98	4.23 ± 1.40	3.93 ± 1.26	4.35 ± 1.14	<0.001^*^
HDL-C, mmol/L	1.53 ± 0.41	1.31 ± 0.42	1.25 ± 0.31	1.34 ± 0.35	<0.001^*^
LDL-C, mmol/L	2.69 ± 0.87	2.36 ± 1.20	2.15 ± 1.04	2.55 ± 1.07	<0.001^*^
ApoA_1_, g/L	1.39 ± 0.28	1.32 ± 0.31	1.25 ± 0.33	1.30 ± 0.29	0.001^*^
ApoB, g/L	0.77 ± 0.27	0.84 ± 0.28	0.81 ± 0.28	0.82 ± 0.31	0.159
Hcy, μmol/L	10.62 ± 3.97	11.11 ± 6.87	12.29 ± 6.12	11.90 ± 4.97	0.003^*^
HHcy, n (%)	8 (9.0)	29 (25.4)	40 (27.6)	22 (21.8)	0.007^*^
NLR	1.79 ± 0.87	2.01 ± 0.87	2.15 ± 1.18	2.31 ± 1.38	0.001^*^
MLR	0.19 ± 0.10	0.17 ± 0.09	0.19 ± 0.10	0.20 ± 0.11	0.094
PLR	126.27 ± 76.03	120.74 ± 51.88	127.39 ± 52.94	144.74 ± 74.85	0.011^*^
SII, 10^9^/L	414.33 ± 289.40	508.17 ± 289.65	569.37 ± 414.91	543.60 ± 448.84	0.001^*^
MHR	0.23 ± 0.11	0.28 ± 0.18	0.29 ± 0.17	0.24 ± 0.16	<0.001^*^

HCs, healthy controls; MMD, moyamoya disease; TIA, transient ischemic attack; SD, standard deviation; SBP, systolic blood pressure; DBP, diastolic blood pressure; BMI, body mass index; IQR, interquartile range; WBC, white blood cell; LY, lymphocyte; RBC, red blood cell; HGB, hemoglobin; HCT, hematocrit; MCV, mean corpuscular volume; MCH, mean corpuscular hemoglobin; MCHC, mean corpuscular hemoglobin concentration; PLT, platelet; TG, triglyceride; TC, total cholesterol; HDL-C, high-density lipoprotein cholesterol; LDL-C, low-density lipoprotein cholesterol; ApoA_1_, apolipoprotein A_1_; ApoB, apolipoprotein B; Hcy, homocysteine; HHcy, hyperhomocysteinemia; NLR, neutrophil-to-lymphocyte ratio; MLR, monocyte-to-lymphocyte ratio; PLR, platelet-to-lymphocyte ratio; SII, systemic immune-inflammation index; MHR, monocyte-to-HDL cholesterol ratio.

* P < 0.05, significant difference.

### Characteristics of MMD patients and HCs according to BCAAs quartiles

Clinical characteristics of MMD patients and HCs according to the BCAAs quartiles were shown in Table 2. Patients with higher level of BCAAs tended to be male; have risk factors of hypertension, diabetes mellitus, hyperlipidemia, cigarette smoking, and alcohol drinking; have higher levels of blood pressure, BMI, WBC count, LY count, RBC, HGB, HCT, glucose, creatinine, uric acid, TG, ApoB, and MHR (P <0.05 for all). Characteristics according to the quartiles of individual BCAAs were summarized in Supplementary Table S1–S3.

**Table 2:** Characteristics of HCs and MMD patients according to BCAAs quartiles.

**Variables**	**Total (*N =* 449)**	**BCAAs quartiles**†, μ**mol/L**				
		**Q1 (*n =* 112)**	**Q2 (*n =* 112)**	**Q3 (*n =* 112)**	**Q4 (*n =* 113)**	***P* for Trend**
Age, y, mean ± SD	41.24 ± 10.53	40.18 ± 10.85	41.79 ± 10.61	41.38 ± 10.13	41.63 ± 10.60	0.378
Sex, male (%)	187 (41.6)	16 (14.3)	37 (33.0)	61 (54.5)	73 (64.6)	<0.001^*^
History of risk factors, n (%)
Hypertension	131 (29.2)	22 (19.6)	26 (23.2)	40 (35.7)	43 (38.1)	<0.001^*^
Diabetes mellitus	59 (13.1)	4 (3.6)	12 (10.7)	10 (8.9)	33 (29.2)	<0.001^*^
Hyperlipidemia	54 (12.0)	9 (8.0)	7 (6.3)	10 (8.9)	28 (24.8)	<0.001^*^
Cigarette smoking	73 (16.3)	5 (4.5)	10 (8.9)	20 (17.9)	38 (33.6)	<0.001^*^
Alcohol drinking	42 (9.4)	1 (0.9)	6 (5.4)	14 (12.5)	21 (18.6)	<0.001^*^
Clinical features, mean ± SD
Heart rate, bpm	78.39 ± 7.19	77.93 ± 6.58	77.21 ± 7.14	79.9 ± 7.92	78.52 ± 6.87	0.139
SBP, mmHg	130.61 ± 13.07	127.77 ± 11.84	129.36 ± 12.10	133.16 ± 14.13	132.12 ± 13.51	0.002^*^
DBP, mmHg	81.18 ± 9.23	80.43 ± 9.01	80.06 ± 8.80	82.06 ± 10.52	82.16 ± 8.41	0.065
BMI, kg/m^2^	25.17 ± 4.35	23.40 ± 3.72	24.84 ± 4.30	25.96 ± 4.53	26.47 ± 4.21	<0.001^*^
Laboratory results, median ± IQR
WBC count, 10^9^/L	6.63 ± 2.27	6.20 ± 2.10	6.51 ± 2.67	6.86 ± 2.53	6.94 ± 2.10	<0.001^*^
LY count, 10^9^/L	1.91 ± 0.82	1.80 ± 0.75	1.82 ± 0.80	1.95 ± 0.80	2.19 ± 0.74	<0.001^*^
Neutrophil count, 10^9^/L	4.07 ± 1.85	3.63 ± 1.58	4.01 ± 1.86	4.29 ± 2.09	4.25 ± 1.82	0.008^*^
Monocyte count, 10^9^/L	0.35 ± 0.16	0.32 ± 0.14	0.34 ± 0.17	0.37 ± 0.13	0.35 ± 0.16	0.014^*^
RBC, 10^12^/L	4.64 ± 0.67	4.41 ± 0.58	4.65 ± 0.73	4.79 ± 0.59	4.86 ± 0.63	<0.001^*^
HGB, g/L	141.00 ± 24.00	134.50 ± 15.00	141.00 ± 23.00	147.00 ± 25.00	149.00 ± 22.00	<0.001^*^
HCT, L/L	0.41 ± 0.07	0.39 ± 0.05	0.41 ± 0.07	0.43 ± 0.07	0.43 ± 0.06	<0.001^*^
MCV, fL	90.00 ± 5.40	90.25 ± 6.20	89.75 ± 5.40	89.65 ± 5.60	89.40 ± 5.00	0.726
MCH, pg	30.80 ± 2.30	30.70 ± 2.70	30.90 ± 1.90	30.80 ± 2.20	30.70 ± 2.10	0.304
MCHC, g/L	342.00 ± 13.00	339.50 ± 12.00	343.00 ± 15.00	343.00 ± 15.00	344.00 ± 12.00	<0.001^*^
PLT count, 10^9^/L	246.00 ± 79.00	249.00 ± 79.00	243.50 ± 87.00	248.50 ± 68.00	238.00 ± 84.00	0.644
Fasting glucose, mmol/L	5.09 ± 0.90	4.95 ± 0.77	5.04 ± 0.80	5.12 ± 0.86	5.27 ± 1.50	<0.001^*^
Creatinine, μmol/L	55.60 ± 20.55	49.65 ± 14.08	53.90 ± 19.02	59.80 ± 19.00	62.90 ± 20.70	<0.001^*^
Uric acid, μmol/L	307.70 ± 115.60	262.15 ± 89.30	292.70 ± 97.80	326.75 ± 99.80	365.50 ± 124.10	<0.001^*^
TG, mmol/L	1.15 ± 0.81	0.90 ± 0.53	1.05 ± 0.78	1.18 ± 0.75	1.44 ± 0.95	<0.001^*^
TC, mmol/L	4.26 ± 1.21	4.34 ± 1.17	4.17 ± 1.36	4.26 ± 1.08	4.31 ± 1.40	0.764
HDL-C, mmol/L	1.34 ± 0.39	1.48 ± 0.45	1.34 ± 0.37	1.33 ± 0.33	1.22 ± 0.35	<0.001^*^
LDL-C, mmol/L	2.41 ± 1.13	2.40 ± 0.99	2.41 ± 1.24	2.42 ± 0.94	2.49 ± 1.23	0.806
ApoA_1_, g/L	1.30 ± 0.29	1.39 ± 0.31	1.33 ± 0.26	1.28 ± 0.27	1.25 ± 0.34	<0.001^*^
ApoB, g/L	0.82 ± 0.27	0.76 ± 0.24	0.76 ± 0.29	0.86 ± 0.24	0.86 ± 0.31	0.002^*^
Hcy, μmol/L	11.43 ± 5.16	10.63 ± 4.54	10.66 ± 4.68	11.85 ± 4.83	12.78 ± 6.21	<0.001^*^
HHcy, n (%)	99 (22.0)	20 (17.9)	18 (16.1)	25 (22.3)	36 (31.9)	0.006^*^
NLR	2.06 ± 1.15	2.01 ± 1.19	2.06 ± 1.18	2.14 ± 1.33	2.01 ± 1.04	0.652
MLR	0.19 ± 0.10	0.19 ± 0.10	0.19 ± 0.09	0.20 ± 0.10	0.18 ± 0.10	0.397
PLR	127.39 ± 58.01	132.14 ± 68.32	137.51 ± 69.92	127.29 ± 54.66	114.72 ± 55.28	0.001^*^
SII, 10^9^/L	505.35 ± 379.14	493.37 ± 439.04	526.63 ± 381.10	577.35 ± 333.42	476.38 ± 330.73	0.851
MHR	0.26 ± 0.16	0.21 ± 0.11	0.25 ± 0.14	0.29 ± 0.16	0.30 ± 0.18	<0.001^*^

HCs, healthy controls; MMD, moyamoya disease; BCAAs, branched-chain amino acids; SD, standard deviation; SBP, systolic blood pressure; DBP, diastolic blood pressure; BMI, body mass index; IQR, interquartile range; WBC, white blood cell; LY, lymphocyte; RBC, red blood cell; HGB, hemoglobin; HCT, hematocrit; MCV, mean corpuscular volume; MCH, mean corpuscular hemoglobin; MCHC, mean corpuscular hemoglobin concentration; PLT, platelet; TG, triglyceride; TC, total cholesterol; HDL-C, high-density lipoprotein cholesterol; LDL-C, low-density lipoprotein cholesterol; ApoA_1_, apolipoprotein A_1_; ApoB, apolipoprotein B; Hcy, homocysteine; HHcy, hyperhomocysteinemia; NLR, neutrophil-to-lymphocyte ratio; MLR, monocyte-to-lymphocyte ratio; PLR, platelet-to-lymphocyte ratio; SII, systemic immune-inflammation index; MHR, monocyte-to-HDL cholesterol ratio.

†Serum levels of BCAAs in quartiles: Q1, <488.9 μmol/L; Q2, 488.9-564.0 μmol/L; Q3, 564.0-639.7 μmol/L; and Q4, ≥ 639.7 μmol/L.

* P < 0.05, significant difference.

### Association of BCAAs with the risk of MMD and its subtypes

**Figure 3:**
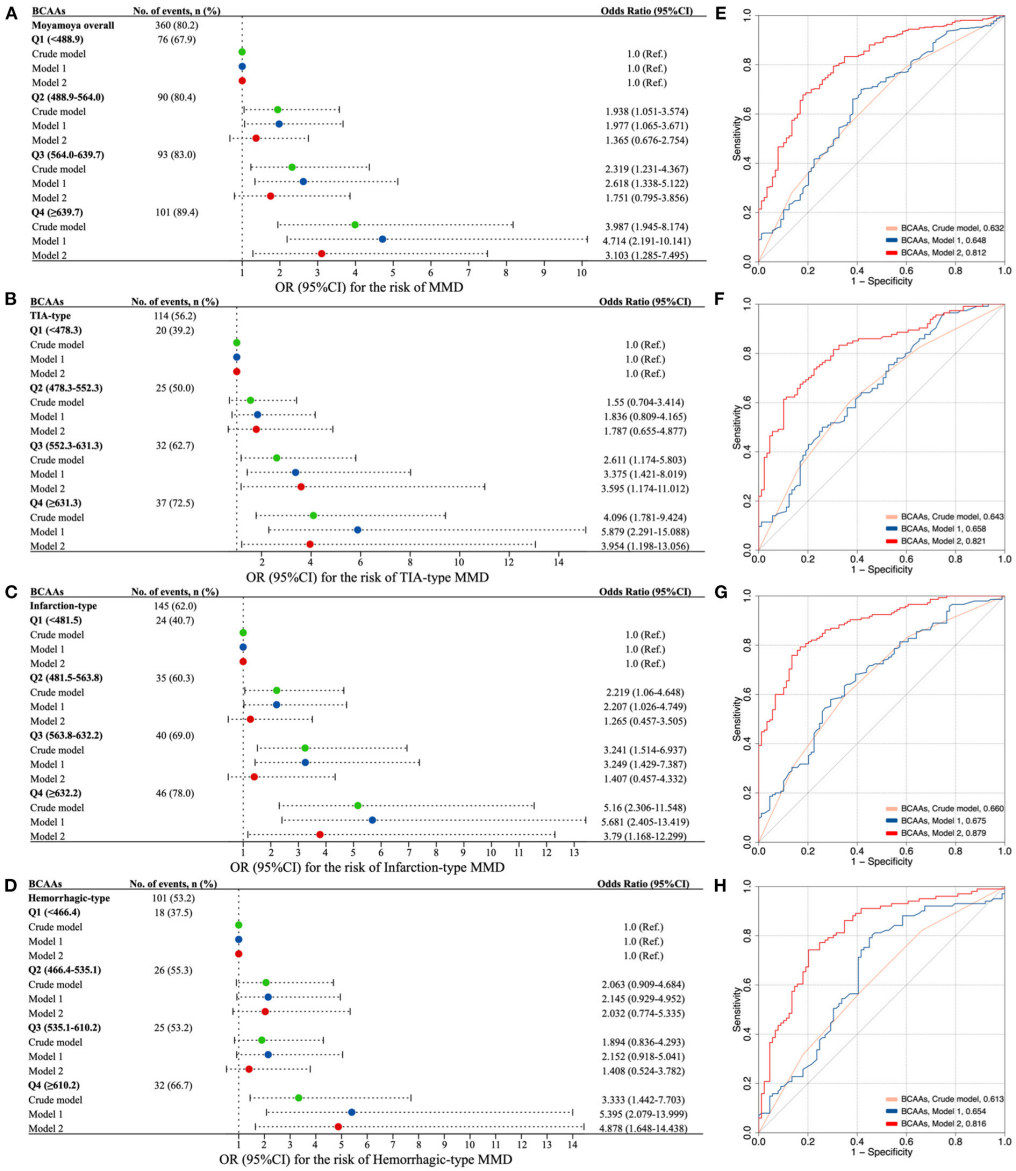
The association of circulating BCAAs level with the risk of MMD and clinical subtypes. **(A–D)** Forest plots for the association of BCAAs with MMD **(A)** and subtypes [**(B)** TIA-type; **(C)** Infarction-type; **(D)** Hemorrhagic-type]. E-H. ROC curves with AUC of different models for the risk of MMD **(E)** and subtypes [**(F)**, TIA-type; **(G)** Infarction-type; **(H)** Hemorrhagic-type]. Model 1, adjusted for age and sex. Model 2, further adjusted for BMI, WBC count, neutrophil count, glucose, TG, TC, HDL-C, LDL-C, APO-A_1_, Hcy, NLR, SII, and MHR. BCAAs, branched-chain amino acids; OR, odds ratio; CI, confidence interval; MMD, moyamoya disease; TIA, transient ischemic attack.

Figure 3 showed the associations of serum total BCAAs with the risk of MMD and its subtypes. The proportion of the presence of MMD in the quartiles of BCAAs increased from 1st to 4th quartiles. After adjusting for age and sex, subjects in the second to last quartiles (Q2–Q4) of BCAAs were associated with a higher risk of MMD than those in the first quartile (Q1). After additionally adjusting for covariates of BMI, WBC count, neutrophil count, glucose, TG, TC, HDL-C, LDL-C, APO-A_1_, Hcy, NLR, SII, and MHR, cases in Q4 of BCAAs were significantly associated with a higher risk of MMD than those in Q1 (odds ratio [OR] 3.10, 95% confidence interval [CI] 1.29-7.50, *P* = 0.012). The ROC curves with AUC of models for the occurrence of MMD were constructed in Figure 3. In contrast to the Crude model and Model 1 (AUC: 0.632, 0.648, respectively), the Model 2 yielded to a prominent improvement in the predictive value (AUC: 0.812).

Consistently, the risk of three subtypes of MMD increased with each increment in the quartiles of BCAAs (Figure 3). Q3 and Q4 of BCAAs were strongly associated with the occurrence TIA-type MMD compared with Q1 in Model 2 (OR 3.60, 95% CI 1.17-11.01, *P* = 0.025; OR 3.95, 95% CI 1.20–13.06, P = 0.024, respectively). Q4 of BCAAs was significantly associated with the risk of infarction-type and hemorrhagic-type MMD compared with Q1 in Model 2 (OR 3.79, 95% CI 1.17–12.30, *P* = 0.027; OR 4.88, 95% CI 1.65–14.44, *P* = 0.004, respectively). In contrast to the Crude model and Model 1, the Model 2 consistently showed prominent improvements in the predictive values of subtypes of TIA, infarction, and hemorrhagic MMD (AUC: 0.821, 0.879, 0.816, respectively) (Figure 3).

Besides, the risk of MMD and its subtypes increased with each increment in the quartiles of individual BCAA (Supplementary Figure S2). Similarly, Q4 of leucine, isoleucine, and valine were markedly associated with the risk of MMD and its subtypes compared with Q1 in Model 2, respectively. The ROC curves with AUC of individual BCAAs models for the presence of MMD and subtypes were constructed in Supplementary Figure S3. Analogously, the predictive values of MMD and its subtypes of Model 2 were all noticeably enhanced, compared with the Crude model and Model 1.

### Improvement in the prediction models for the risk of MMD and its subtypes

We compared the performance of different models for predicting the risk of MMD and its subtypes (Table 3). The addition of BCAAs to the basic model moderately improved the performance verified by NRI. The NRI of BCAAs in quartiles for the presence of MMD was 35.4% (95%CI 13.2–57.6%). A similar performance for predicting the risk of MMD was validated for BCAAs and the basic model. The IDI of BCAAs in quartiles for the occurrence of MMD was 1.7% (95%CI 0.3–3.2%). In addition, the predictive performance of BCAAs in MMD subtypes evaluated by NRI and IDI was consistent. Significant improvements were also observed after the addition of BCAAs to the basic model by NRI and IDI.

**Table 3:** Performance of models with BCAAs to predict the risk of MMD and its subtypes.

**Variables†**	**NRI, continuous**		**IDI**	
	**Estimate (95%CI), %**	***P* value**	**Estimate (95%CI), %**	***P* value**
**Moyamoya overall**				
Basic model	Ref.		Ref.	
Basic model + BCAAs quartiles	35.4 (13.2–57.6)	0.002^*^	1.7 (0.3–3.2)	0.022^*^
Basic model + BCAAs continuous	35.2 (12.4–58.0)	0.002^*^	2.1 (0.5–3.7)	0.011^*^
**TIA-type**				
Basic model	Ref.		Ref.	
Basic model + BCAAs quartiles	38.7 (11.5–65.9)	0.005^*^	2.6 (0.3–4.9)	0.027^*^
Basic model + BCAAs continuous	41.5 (14.5–68.6)	0.003^*^	2.4 (0.2–4.7)	0.036^*^
**Infarction-type**				
Basic model	Ref.		Ref.	
Basic model + BCAAs quartiles	37.6 (12.9–62.4)	0.003^*^	2.0 (0.1–3.8)	0.038^*^
Basic model + BCAAs continuous	24.1 (−1.9–50.1)	0.069	2.2 (0.3–4.1)	0.023^*^
**Hemorrhagic-type**				
Basic model	Ref.		Ref.	
Basic model + BCAAs quartiles	48.2 (20.7–75.7)	<0.001^*^	4.6 (1.7–7.5)	0.002^*^
Basic model + BCAAs continuous	40.6 (12.8–68.5)	0.004^*^	2.7 (0.6–4.8)	0.013^*^

BCAAs, branched-chain amino acids; MMD, moyamoya disease; NRI, net reclassification index; IDI, integrated discrimination improvement; CI, confidence interval; TIA, transient ischemic attack.

†Basic model included age, sex, BMI, WBC count, Neutrophil count, glucose, TG, TC, HDL-C, LDL-C, ApoA_1_, Hcy, NLR, SII, and MHR.

* P < 0.05, significant difference.

## Discussion

In this large case-control study of 449 participants, we firstly investigated the association of serum metabolite of BCAAs with the risk of MMD and clinical subtypes. We identified that the serum level of BCAAs was significantly higher in MMD patients than in HCs. The elevated level of BCAAs was strongly associated with increased risks of MMD and subtypes. Collectively, our findings outlined the crucial relevance of increasing serum BCAAs with the risk of MMD.

Amino acids are important nutrients for humans. As the precursors of proteins, amino acids participate in various life activities and metabolism (13). BCAAs is an essential amino acid that regulates cell growth, autophagy, neurotransmitter synthesis, carbohydrate and lipid metabolism (14). The excessive intake of amino acids and metabolic disorders of BCAAs would result in the accumulation of serum BCAAs, which have been verified in animal experiments and clinical studies (15, 16). Therefore, the increment of BCAAs is often considered as the evidence of metabolic disorders. Current studies have confirmed that BCAAs is associated with diabetes, obesity, insulin resistance and other diseases (7). The metabolites of BCAA pathway accumulate as the risk factor for insulin resistance, and the association was identified in type 2 diabetes and cardiovascular disease (7, 17). Previous studies have shown that abnormal metabolism of BCAAs was associated with a variety of cardio-cerebrovascular diseases, including coronary heart disease, heart failure, and carotid artery stenosis (18–20). Few metabolomics studies have been conducted in patients with MMD (11, 12). The studies consistently demonstrated that the level of serum valine in MMD patients was significantly lower than that in HCs, while the result of isoleucine was quite the opposite. It seems possible that the inverse result of valine is due to the different technology of mass spectrometry used for the serum metabolome. In general, our findings indicated that the altered levels of BCAAs could be linked to MMD.

As nutrient signaling molecules, BCAAs mainly transduce the mTOR pathway (8). BCAAs, especially leucine, participate in many biological activities by activating mTOR. One study showed that isoleucine in mitochondria was involved in the vascular oxidative stress, leading to the endothelial dysfunction (21). MMD is a multifactorial disease, affected by genetic and environmental factors (22). Various risk factors can cause an elevation of free radicals, continuingly producing excessive reactive oxygen species (ROS) that result in cellular damage (23). Jung et al. found that the oxidative stress level of endothelial colony-forming cells (ECFCs) in patients with MMD was significantly higher than in HCs (24). The angiogenesis capacity of endothelial cells in MMD patients was increased by administrating the ROS scavengers. Endothelial cells are prone to have oxidative stress response and generate various biologically active substances by exposed to the microenvironment in plasma, causing a functional impairment in endothelial cells through various pathways. Therefore, we hypothesized that high concentration of BCAAs could induce the activation of mTOR, resulting in oxidative stress, mitochondrial dysfunction, and apoptosis, which may be one of the mechanisms involved in the pathogenesis of MMD.

BCAAs may generate a chronic inflammatory response by increasing the expression of pro-inflammatory cytokines (e.g., TNF-α and IL-6), leading to changes in endothelial and smooth muscle cell phenotypes, and thereby producing the pathological conditions. Some studies have identified that isoleucine was positively correlated with IL-6, endotoxin and oxLDL, (25) while leucine was positively related to TNF-α and HOMA-IR (26). The supplementation of BCAAs in blood monocytes stimulated redox through NADPH oxidase and the generation of ROS throughout the mitochondria activation of NF-κB, resulting in the release of pro-inflammatory factors (27). Recent studies have found that the expression levels of periphery inflammatory factors in MMD were higher than those in HCs, including TNF-α, IL1-β, IL-6, etc. (28, 29). The microenvironment formed by abnormally secreted inflammatory factors may promote the proliferation and angiogenesis of cells in affected vessels (30). The occurrence of chronic inflammation may generate the vascular damage and the formation of micro-vessels, leading to the hemorrhage and infarction (31). In conclusion, BCAAs may have an impact on endothelial and smooth muscle cells through oxidative stress and inflammatory responses, and thereby develop the phenotype of moyamoya vasculopathy.

Our study showed that several factors were associated with the quartiles of BCAAs. There were growing trends of diabetes and BMI along with the level of BCAAs. It has been verified that BCAAs was related to the metabolic disorders, including diabetes and obesity (7). Although diabetes and obesity are comorbidities in patients with MMD (2), BCAAs is still the independent risk factor after adjustment for glucose and BMI in the multivariate regression models. In addition, we confirmed that the TG level were positively correlated with BCAAs. BCAAs exert an influence on the lipid metabolism (32). It is logically consistent with our previous case-control study which has shown that the dyslipidemia was linked to MMD (5). We also detected that the elevated levels of hematologic indicators (RBC, HGB, HCT) were in parallel with the increment of BCAAs. Recent study has shown that BCAAs was related to the iron metabolism, and the circulating BCAAs was decreased in patients with anemia (33). Therefore, we concluded that the disorder of BCAAs may serve as a bridge connecting multiple metabolic abnormalities and diseases.

In this study, total and individual BCAAs were all analyzed. The differences among three individual BCAAs tended to be similar. Although BCAAs is a compound of three substances, each BCAA may have distinct effects. Isoleucine and valine, while not leucine, mediated the metabolic health (34). It is only isoleucine, not leucine and valine, that improved the brain perfusion (35). In addition, we found that circulating BCAAs in MMD patients is higher than that of HCs. The consequence of significant difference is the initial trigger of onset or the result of second strike cannot be clarified based on the current case-control study. Hence, it is vital to demonstrate the function of each BCAA metabolite in MMD.

Several limitations should be considered in this study. First, this is a single-center study with relatively small sample size. Although the potential bias was inevitable, this is the largest study to investigate the association of serum BCAAs and MMD. Second, the study was conducted in a Chinese population with adult MMD, the findings may not be generalized to the overall populations of MMD. Third, the information of patient-level diets was not included in the study. The pattern of diets may have an impact on the outcomes. Fourth, the results of serum metabolites are affected by many factors. Although the confounders have been adjusted in the regression models, we can only demonstrate the association of serum BCAAs with the risk of MMD. Further *in vitro* or *in vivo* experiments, and larger prospective cohort studies with follow-up outcomes are warranted to reveal the effect and mechanism of BCAAs on the pathogenesis of MMD.

## Conclusions

Our study indicated that higher circulating BCAAs level was associated with increased risk of MMD and clinical subtypes. This work will help to elucidate the pathogenesis of MMD, which may provide the support for facilitating the interventions and preventions.

## Data availability statement

The raw data supporting the conclusions of this article will be made available by the authors, without undue reservation.

## Ethics statement

The studies involving human participants were reviewed and approved by Beijing Tiantan Hospital, Capital Medical University. The patients/participants provided their written informed consent to participate in this study.

## Author contributions

Study concept and design: CZ and PG. Collection and assembly of data: CZ, PG, CL, XYu, and YZhai. Data analysis and interpretation: WL, QH, JL, XL, and JW. Manuscript writing: CZ. Manuscript revision: XYe, QZ, RW, and YZhang. Provision of study materials: JZ and DZ. Final approval of manuscript: all authors.

## Funding

This study was supported by National Key Research and Development Program of China (2021YFC2500502), National Natural Science Foundation of China (81701137 and 81870904), Beijing Municipal Commission of Education (KM201910025014), and Beijing Municipal Administration of Hospitals' Mission Plan (SML20150501).

## Conflict of interest

The authors declare that the research was conducted in the absence of any commercial or financial relationships that could be construed as a potential conflict of interest.

## Publisher's note

All claims expressed in this article are solely those of the authors and do not necessarily represent those of their affiliated organizations, or those of the publisher, the editors and the reviewers. Any product that may be evaluated in this article, or claim that may be made by its manufacturer, is not guaranteed or endorsed by the publisher.
