# Supplementary material for: Association of circulating branched-chain amino acids with risk of moyamoya disease
Source: Front Nutr. 2022 Sep 2;9:994286. doi: 10.3389/fnut.2022.994286 (PMC9479188; doi:10.3389/fnut.2022.994286)
Supplement: Supplementary file 1 [file Data_Sheet_1.PDF]

Supplementary Material

Association of Circulating Branched-Chain Amino Acids with Risk of Moyamoya Disease

Chaofan Zeng[1-5†], Peicong Ge[1-5†], Chenglong Liu[1-5], Xiaofan Yu[1-5], Yuanren Zhai[1-5], Wei Liu[1-5], Qiheng He[1-5], Junsheng Li[1-5], Xingju Liu[1-5], Jia Wang[1-5], Xun Ye[1-5], Qian Zhang[1-5], Rong Wang[1-5], Yan Zhang[1-5], Jizong Zhao[1-5*], Dong Zhang[1-6*]

[1]Department of Neurosurgery, Beijing Tiantan Hospital, Capital Medical University, Beijing, China

[2]China National Clinical Research Center for Neurological Diseases, Beijing, China

[3]Center of Stroke, Beijing Institute for Brain Disorders, Beijing, China

[4]Beijing Key Laboratory of Translational Medicine for Cerebrovascular Disease, Beijing, China

[5]Beijing Translational Engineering Center for 3D Printer in Clinical Neuroscience, Beijing, China

[6]Department of Neurosurgery, Beijing Hospital, Beijing, China

[†]These authors contributed equally to this work and share first authorship

***Corresponding Author:**

Jizong Zhao, Department of Neurosurgery, Beijing Tiantan Hospital, Capital Medical University, Beijing, 100070, China, Email: zhaojizong@bjtth.org

Dong Zhang, Department of Neurosurgery, Beijing Tiantan Hospital, Capital Medical University, Beijing, 100070, China, Email: zhangdong0660@aliyun.com

Table S1. Characteristics of HCs and MMD patients according to Leucine quartiles

Variables	Total (N=449)	Leucine quartiles†, μmol/L				
		Q1 (n=112)	Q2 (n=112)	Q3 (n=112)	Q4 (n=113)	*P* for Trend
Age, y, mean ± SD	41.24 ± 10.53	40.44 ± 10.84	41.67 ± 10.50	41.67 ± 10.24	41.20 ± 10.64	0.606
Sex, male (%)	187 (41.6)	17 (15.2)	28 (25.0)	64 (57.1)	78 (69.0)	< 0.001*
History of risk factors, n (%)						
Hypertension	131 (29.2)	20 (17.9)	28 (25.0)	45 (40.2)	38 (33.6)	0.001*
Diabetes mellitus	59 (13.1)	6 (5.4)	8 (7.1)	16 (14.3)	29 (25.7)	< 0.001*
Hyperlipidemia	54 (12.0)	8 (7.1)	6 (5.4)	18 (16.1)	22 (19.5)	0.001*
Cigarette smoking	73 (16.3)	4 (3.6)	9 (8.0)	21 (18.8)	39 (34.5)	< 0.001*
Alcohol drinking	42 (9.4)	1 (0.9)	5 (4.5)	17 (15.2)	19 (16.8)	< 0.001*
Clinical features, mean ± SD						
Heart rate, bpm	78.39 ± 7.19	77.43 ± 6.46	78.00 ± 6.87	78.93 ± 7.82	79.20 ± 7.48	0.039*
SBP, mmHg	130.61 ± 13.07	127.04 ± 12.01	130.14 ± 13.36	132.29 ± 13.49	132.92 ± 12.72	< 0.001*
DBP, mmHg	81.18 ± 9.23	79.29 ± 8.90	81.11 ± 9.62	80.94 ± 9.97	83.36 ± 8.01	0.002*
BMI, kg/m^2	25.17 ± 4.35	23.83 ± 4.57	24.44 ± 3.90	25.83 ± 3.94	26.57 ± 4.45	< 0.001*
Laboratory results, median ± IQR						
WBC count, 10^9/L	6.63 ± 2.27	6.03 ± 2.31	6.43 ± 2.17	7.05 ± 2.55	6.94 ± 2.11	< 0.001*
LY count, 10^9/L	1.91 ± 0.82	1.67 ± 0.66	1.89 ± 0.67	1.98 ± 0.86	2.17 ± 0.75	< 0.001*
Neutrophil count, 10^9/L	4.07 ± 1.85	3.64 ± 1.64	4.08 ± 1.81	4.24 ± 1.99	4.19 ± 1.88	0.006*
Monocyte count, 10^9/L	0.35 ± 0.16	0.32 ± 0.14	0.33 ± 0.15	0.38 ± 0.16	0.36 ± 0.16	< 0.001*
RBC, 10^{12}/L	4.64 ± 0.67	4.45 ± 0.67	4.58 ± 0.54	4.74 ± 0.65	4.92 ± 0.58	< 0.001*
HGB, g/L	141.00 ± 24.00	133.00 ± 18.00	138.50 ± 20.00	146.00 ± 25.00	153.00 ± 19.00	< 0.001*
HCT, L/L	0.41 ± 0.07	0.40 ± 0.05	0.41 ± 0.05	0.42 ± 0.07	0.44 ± 0.05	< 0.001*
MCV, fL	90.00 ± 5.40	90.20 ± 6.10	88.90 ± 5.80	90.20 ± 6.20	90.00 ± 4.50	0.045*
MCH, pg	30.80 ± 2.30	30.80 ± 2.70	30.60 ± 2.80	30.90 ± 2.40	30.70 ± 2.10	< 0.001*
MCHC, g/L	342.00 ± 13.00	339.00 ± 14.00	342.00 ± 14.00	343.00 ± 14.00	344.00 ± 11.00	< 0.001*
PLT count, 10^9/L	246.00 ± 79.00	245.50 ± 78.00	251.50 ± 86.00	241.50 ± 75.00	246.00 ± 76.00	0.926
Fasting glucose, mmol/L	5.09 ± 0.90	4.99 ± 0.74	5.03 ± 0.87	5.13 ± 0.92	5.23 ± 1.46	< 0.001*
Creatinine, μmol/L	55.60 ± 20.55	50.05 ± 14.20	51.35 ± 13.20	63.55 ± 20.20	63.20 ± 19.10	< 0.001*
Uric acid, μmol/L	307.70 ± 115.60	256.05 ± 101.70	287.95 ± 77.00	335.15 ± 104.00	365.50 ± 117.90	< 0.001*
TG, mmol/L	1.15 ± 0.81	0.85 ± 0.55	1.06 ± 0.77	1.22 ± 0.71	1.44 ± 0.92	< 0.001*
TC, mmol/L	4.26 ± 1.21	4.23 ± 1.20	4.26 ± 1.25	4.24 ± 1.32	4.36 ± 1.26	0.672
HDL-C, mmol/L	1.34 ± 0.39	1.49 ± 0.43	1.33 ± 0.34	1.32 ± 0.41	1.25 ± 0.33	< 0.001*
LDL-C, mmol/L	2.41 ± 1.13	2.30 ± 1.02	2.48 ± 1.08	2.44 ± 1.14	2.46 ± 1.15	0.370
$ApoA_1$, g/L	1.30 ± 0.29	1.40 ± 0.30	1.30 ± 0.28	1.30 ± 0.30	1.23 ± 0.32	< 0.001*
ApoB, g/L	0.82 ± 0.27	0.74 ± 0.26	0.79 ± 0.29	0.84 ± 0.24	0.87 ± 0.30	< 0.001*
Hcy, μmol/L	11.43 ± 5.16	10.60 ± 4.42	10.37 ± 4.84	11.75 ± 4.94	12.78 ± 6.20	< 0.001*

HHcy, n (%)	99 (22.0)	17 (15.0)	21 (18.8)	24 (21.4)	37 (32.7)	0.002*
NLR	2.06 ± 1.15	2.03 ± 1.28	2.05 ± 1.06	2.13 ± 1.31	1.94 ± 1.00	0.411
MLR	0.19 ± 0.10	0.20 ± 0.10	0.18 ± 0.10	0.20 ± 0.10	0.18 ± 0.09	0.681
PLR	127.39 ± 58.01	137.23 ± 77.72	139.04 ± 75.15	120.11 ± 54.72	114.24 ± 52.93	< 0.001*
SII, 10^9/L	505.35 ± 379.14	507.42 ± 426.61	527.32 ± 410.81	549.30 ± 374.09	473.88 ± 322.10	0.561
MHR	0.26 ± 0.16	0.22 ± 0.11	0.24 ± 0.14	0.31 ± 0.18	0.30 ± 0.17	< 0.001*

HCs, healthy controls; MMD, moyamoya disease; SD, standard deviation; SBP, systolic blood pressure; DBP, diastolic blood pressure; BMI, body mass index; IQR, interquartile range; WBC, white blood cell; LY, lymphocyte; RBC, red blood cell; HGB, hemoglobin; HCT, hematocrit; MCV, mean corpuscular volume; MCH, mean corpuscular hemoglobin; MCHC, mean corpuscular hemoglobin concentration; PLT, platelet; TG, triglyceride; TC, total cholesterol; HDL-C, high-density lipoprotein cholesterol; LDL-C, low-density lipoprotein cholesterol; $ApoA_1$, apolipoprotein A_1; ApoB, apolipoprotein B; Hcy, homocysteine; HHcy, hyperhomocysteinemia; NLR, neutrophil-to-lymphocyte ratio; MLR, monocyte-to-lymphocyte ratio; PLR, platelet-to-lymphocyte ratio; SII, systemic immune-inflammation index; MHR, monocyte-to-HDL cholesterol ratio.

†Serum levels of Leucine in quartiles: Q1, < 181.4 μmol/L; Q2, 181.4-212.8 μmol/L; Q3, 212.8-242.2 μmol/L; and Q4, ≥ 242.2 μmol/L.

*P＜0.05, significant difference.

Table S2. Characteristics of HCs and MMD patients according to Isoleucine quartiles

Variables	Total (N=449)	Isoleucine quartiles†, μmol/L				
		Q1 (n=112)	Q2 (n=112)	Q3 (n=112)	Q4 (n=113)	*P* for Trend
Age, y, mean ± SD	41.24 ± 10.53	40.44 ± 10.84	41.67 ± 10.50	41.67 ± 10.24	41.20 ± 10.64	0.606
Sex, male (%)	187 (41.6)	17 (15.2)	28 (25.0)	64 (57.1)	78 (69.0)	< 0.001*
History of risk factors, n (%)						
Hypertension	131 (29.2)	20 (17.9)	28 (25.0)	45 (40.2)	38 (33.6)	0.001*
Diabetes mellitus	59 (13.1)	6 (5.4)	8 (7.1)	16 (14.3)	29 (25.7)	< 0.001*
Hyperlipidemia	54 (12.0)	8 (7.1)	6 (5.4)	18 (16.1)	22 (19.5)	0.001*
Cigarette smoking	73 (16.3)	4 (3.6)	9 (8.0)	21 (18.8)	39 (34.5)	< 0.001*
Alcohol drinking	42 (9.4)	1 (0.9)	5 (4.5)	17 (15.2)	19 (16.8)	< 0.001*
Clinical features, mean ± SD						
Heart rate, bpm	78.39 ± 7.19	77.43 ± 6.46	78.00 ± 6.87	78.93 ± 7.82	79.20 ± 7.48	0.039*
SBP, mmHg	130.61 ± 13.07	127.04 ± 12.01	130.14 ± 13.36	132.29 ± 13.49	132.92 ± 12.72	< 0.001*
DBP, mmHg	81.18 ± 9.23	79.29 ± 8.90	81.11 ± 9.62	80.94 ± 9.97	83.36 ± 8.01	0.002*
BMI, kg/m^2	25.17 ± 4.35	23.83 ± 4.57	24.44 ± 3.90	25.83 ± 3.94	26.57 ± 4.45	< 0.001*
Laboratory results, median ± IQR						
WBC count, 10^9/L	6.63 ± 2.27	6.03 ± 2.31	6.43 ± 2.17	7.05 ± 2.55	6.94 ± 2.11	< 0.001*
LY count, 10^9/L	1.91 ± 0.82	1.67 ± 0.66	1.89 ± 0.67	1.98 ± 0.86	2.17 ± 0.75	< 0.001*
Neutrophil count, 10^9/L	4.07 ± 1.85	3.64 ± 1.64	4.08 ± 1.81	4.24 ± 1.99	4.19 ± 1.88	0.006*
Monocyte count, 10^9/L	0.35 ± 0.16	0.32 ± 0.14	0.33 ± 0.15	0.38 ± 0.16	0.36 ± 0.16	< 0.001*
RBC, 10^{12}/L	4.64 ± 0.67	4.45 ± 0.67	4.58 ± 0.54	4.74 ± 0.65	4.92 ± 0.58	< 0.001*
HGB, g/L	141.00 ± 24.00	133.00 ± 18.00	138.50 ± 20.00	146.00 ± 25.00	153.00 ± 19.00	< 0.001*
HCT, L/L	0.41 ± 0.07	0.40 ± 0.05	0.41 ± 0.05	0.42 ± 0.07	0.44 ± 0.05	< 0.001*
MCV, fL	90.00 ± 5.40	90.20 ± 6.10	88.90 ± 5.80	90.20 ± 6.20	90.00 ± 4.50	0.045*
MCH, pg	30.80 ± 2.30	30.80 ± 2.70	30.60 ± 2.80	30.90 ± 2.40	30.70 ± 2.10	< 0.001*
MCHC, g/L	342.00 ± 13.00	339.00 ± 14.00	342.00 ± 14.00	343.00 ± 14.00	344.00 ± 11.00	< 0.001*
PLT count, 10^9/L	246.00 ± 79.00	245.50 ± 78.00	251.50 ± 86.00	241.50 ± 75.00	246.00 ± 76.00	0.926
Fasting glucose, mmol/L	5.09 ± 0.90	4.99 ± 0.74	5.03 ± 0.87	5.13 ± 0.92	5.23 ± 1.46	< 0.001*
Creatinine, μmol/L	55.60 ± 20.55	50.05 ± 14.20	51.35 ± 13.20	63.55 ± 20.20	63.20 ± 19.10	< 0.001*
Uric acid, μmol/L	307.70 ± 115.60	256.05 ± 101.70	287.95 ± 77.00	335.15 ± 104.00	365.50 ± 117.90	< 0.001*
TG, mmol/L	1.15 ± 0.81	0.85 ± 0.55	1.06 ± 0.77	1.22 ± 0.71	1.44 ± 0.92	< 0.001*
TC, mmol/L	4.26 ± 1.21	4.23 ± 1.20	4.26 ± 1.25	4.24 ± 1.32	4.36 ± 1.26	0.672
HDL-C, mmol/L	1.34 ± 0.39	1.49 ± 0.43	1.33 ± 0.34	1.32 ± 0.41	1.25 ± 0.33	< 0.001*
LDL-C, mmol/L	2.41 ± 1.13	2.30 ± 1.02	2.48 ± 1.08	2.44 ± 1.14	2.46 ± 1.15	0.370
$ApoA_1$, g/L	1.30 ± 0.29	1.40 ± 0.30	1.30 ± 0.28	1.30 ± 0.30	1.23 ± 0.32	< 0.001*
ApoB, g/L	0.82 ± 0.27	0.74 ± 0.26	0.79 ± 0.29	0.84 ± 0.24	0.87 ± 0.30	< 0.001*
Hcy, μmol/L	11.43 ± 5.16	10.60 ± 4.42	10.37 ± 4.84	11.75 ± 4.94	12.78 ± 6.20	< 0.001*

HHcy, n (%)	99 (22.0)	17 (15.0)	21 (18.8)	24 (21.4)	37 (32.7)	0.002*
NLR	2.06 ± 1.15	2.03 ± 1.28	2.05 ± 1.06	2.13 ± 1.31	1.94 ± 1.00	0.411
MLR	0.19 ± 0.10	0.20 ± 0.10	0.18 ± 0.10	0.20 ± 0.10	0.18 ± 0.09	0.681
PLR	127.39 ± 58.01	137.23 ± 77.72	139.04 ± 75.15	120.11 ± 54.72	114.24 ± 52.93	< 0.001*
SII, 10^9/L	505.35 ± 379.14	507.42 ± 426.61	527.32 ± 410.81	549.30 ± 374.09	473.88 ± 322.10	0.561
MHR	0.26 ± 0.16	0.22 ± 0.11	0.24 ± 0.14	0.31 ± 0.18	0.30 ± 0.17	< 0.001*

HCs, healthy controls; MMD, moyamoya disease; SD, standard deviation; SBP, systolic blood pressure; DBP, diastolic blood pressure; BMI, body mass index; IQR, interquartile range; WBC, white blood cell; LY, lymphocyte; RBC, red blood cell; HGB, hemoglobin; HCT, hematocrit; MCV, mean corpuscular volume; MCH, mean corpuscular hemoglobin; MCHC, mean corpuscular hemoglobin concentration; PLT, platelet; TG, triglyceride; TC, total cholesterol; HDL-C, high-density lipoprotein cholesterol; LDL-C, low-density lipoprotein cholesterol; $ApoA_1$, apolipoprotein A_1; ApoB, apolipoprotein B; Hcy, homocysteine; HHcy, hyperhomocysteinemia; NLR, neutrophil-to-lymphocyte ratio; MLR, monocyte-to-lymphocyte ratio; PLR, platelet-to-lymphocyte ratio; SII, systemic immune-inflammation index; MHR, monocyte-to-HDL cholesterol ratio.

†Serum levels of Isoleucine in quartiles: Q1, < 81.5 μmol/L; Q2, 81.5-95.8 μmol/L; Q3, 95.8-111.5 μmol/L; and Q4, ≥ 111.5 μmol/L.

*P＜0.05, significant difference.

Table S3. Characteristics of HCs and MMD patients according to Valine quartiles

Variables	Total (N=449)	Valine quartiles†, µmol/L				
		Q1 (n=112)	Q2 (n=112)	Q3 (n=112)	Q4 (n=113)	*P* for Trend
Age, y, mean ± SD	41.24 ± 10.53	39.73 ± 10.47	40.79 ± 10.64	42.21 ± 10.76	42.24 ± 10.21	0.045*
Sex, male (%)	187 (41.6)	25 (22.3)	37 (33.0)	53 (47.3)	72 (63.7)	< 0.001*
History of risk factors, n (%)						
Hypertension	131 (29.2)	23 (20.5)	29 (25.9)	34 (30.4)	45 (39.8)	0.001*
Diabetes mellitus	59 (13.1)	8 (7.1)	9 (8.0)	13 (11.6)	29 (25.7)	< 0.001*
Hyperlipidemia	54 (12.0)	9 (8.0)	7 (6.3)	14 (12.5)	24 (21.2)	0.001*
Cigarette smoking	73 (16.3)	7 (6.3)	12 (10.7)	21 (18.8)	33 (29.2)	< 0.001*
Alcohol drinking	42 (9.4)	2 (1.8)	6 (5.4)	15 (13.4)	19 (16.8)	< 0.001*
Clinical features, mean ± SD						
Heart rate, bpm	78.39 ± 7.19	77.1 ± 6.70	78.46 ± 6.87	78.88 ± 7.76	79.13 ± 7.30	0.032*
SBP, mmHg	130.61 ± 13.07	127.12 ± 11.80	130.56 ± 10.89	132.77 ± 16.09	131.96 ± 12.33	0.002*
DBP, mmHg	81.18 ± 9.23	80.34 ± 9.02	79.96 ± 8.29	82.43 ± 11.06	81.98 ± 8.17	0.058*
BMI, kg/m^2	25.17 ± 4.35	23.93 ± 4.63	24.53 ± 3.46	25.79 ± 4.70	26.42 ± 4.10	< 0.001*
Laboratory results, median ± IQR						
WBC count, 10^9/L	6.63 ± 2.27	6.24 ± 2.34	6.56 ± 2.42	6.79 ± 2.49	6.94 ± 2.22	0.002*
LY count, 10^9/L	1.91 ± 0.82	1.75 ± 0.78	1.90 ± 0.74	1.89 ± 0.74	2.23 ± 0.78	0.001*
Neutrophil count, 10^9/L	4.07 ± 1.85	3.69 ± 1.75	4.19 ± 1.94	4.16 ± 1.86	4.19 ± 1.98	0.034*
Monocyte count, 10^9/L	0.35 ± 0.16	0.33 ± 0.15	0.35 ± 0.15	0.36 ± 0.15	0.36 ± 0.15	0.092
RBC, 10^{12}/L	4.64 ± 0.67	4.46 ± 0.69	4.58 ± 0.68	4.74 ± 0.59	4.88 ± 0.60	< 0.001*
HGB, g/L	141.00 ± 24.00	135.00 ± 17.00	140.50 ± 22.00	146.00 ± 22.00	149.00 ± 22.00	< 0.001*
HCT, L/L	0.41 ± 0.07	0.40 ± 0.05	0.41 ± 0.06	0.42 ± 0.06	0.44 ± 0.05	< 0.001*
MCV, fL	90.00 ± 5.40	90.20 ± 6.30	89.70 ± 5.40	89.80 ± 5.70	89.80 ± 5.10	0.117
MCH, pg	30.80 ± 2.30	30.75 ± 2.60	30.85 ± 2.20	30.80 ± 2.30	30.70 ± 1.90	0.007*
MCHC, g/L	342.00 ± 13.00	339.00 ± 14.00	343.00 ± 14.00	343.00 ± 14.00	343.00 ± 11.00	< 0.001*
PLT count, 10^9/L	246.00 ± 79.00	247.00 ± 78.00	251.00 ± 90.00	246.00 ± 71.00	237.00 ± 79.00	0.960
Fasting glucose, mmol/L	5.09 ± 0.90	5.01 ± 0.82	5.02 ± 0.67	5.09 ± 1.00	5.25 ± 1.24	< 0.001*
Creatinine, µmol/L	55.60 ± 20.55	50.45 ± 16.70	54.20 ± 17.35	58.60 ± 21.45	62.50 ± 20.20	< 0.001*
Uric acid, µmol/L	307.70 ± 115.60	265.45 ± 104.30	299.50 ± 87.10	322.85 ± 110.20	358.20 ± 132.40	< 0.001*
TG, mmol/L	1.15 ± 0.81	0.89 ± 0.52	1.11 ± 0.72	1.19 ± 0.70	1.46 ± 0.90	< 0.001*
TC, mmol/L	4.26 ± 1.21	4.23 ± 1.13	4.32 ± 1.32	4.25 ± 1.25	4.31 ± 1.30	0.347
HDL-C, mmol/L	1.34 ± 0.39	1.45 ± 0.36	1.34 ± 0.39	1.34 ± 0.40	1.26 ± 0.32	< 0.001*
LDL-C, mmol/L	2.41 ± 1.13	2.30 ± 1.02	2.48 ± 1.14	2.41 ± 1.05	2.49 ± 1.18	0.225
$ApoA_1$, g/L	1.30 ± 0.29	1.36 ± 0.32	1.29 ± 0.25	1.31 ± 0.37	1.29 ± 0.33	0.010*
ApoB, g/L	0.82 ± 0.27	0.75 ± 0.26	0.78 ± 0.29	0.85 ± 0.28	0.86 ± 0.29	< 0.001*
Hcy, µmol/L	11.43 ± 5.16	11.05 ± 5.58	10.68 ± 5.00	11.14 ± 4.20	12.78 ± 6.40	0.018*

HHcy, n (%)	99 (22.0)	22 (19.6)	24 (21.4)	16 (14.3)	37 (32.7)	0.065
NLR	2.06 ± 1.15	2.03 ± 1.34	1.95 ± 1.16	2.20 ± 1.11	1.94 ± 1.00	0.786
MLR	0.19 ± 0.10	0.19 ± 0.10	0.19 ± 0.10	0.19 ± 0.10	0.17 ± 0.10	0.427
PLR	127.39 ± 58.01	130.03 ± 70.13	137.89 ± 77.63	130.90 ± 52.30	114.72 ± 55.69	0.003*
SII, 10^9/L	505.35 ± 379.14	496.68 ± 444.35	514.76 ± 385.68	558.51 ± 432.03	476.38 ± 296.23	0.832
MHR	0.26 ± 0.16	0.22 ± 0.15	0.26 ± 0.15	0.29 ± 0.15	0.29 ± 0.18	< 0.001*

HCs, healthy controls; MMD, moyamoya disease; SD, standard deviation; SBP, systolic blood pressure; DBP, diastolic blood pressure; BMI, body mass index; IQR, interquartile range; WBC, white blood cell; LY, lymphocyte; RBC, red blood cell; HGB, hemoglobin; HCT, hematocrit; MCV, mean corpuscular volume; MCH, mean corpuscular hemoglobin; MCHC, mean corpuscular hemoglobin concentration; PLT, platelet; TG, triglyceride; TC, total cholesterol; HDL-C, high-density lipoprotein cholesterol; LDL-C, low-density lipoprotein cholesterol; $ApoA_1$, apolipoprotein A_1; ApoB, apolipoprotein B; Hcy, homocysteine; HHcy, hyperhomocysteinemia; NLR, neutrophil-to-lymphocyte ratio; MLR, monocyte-to-lymphocyte ratio; PLR, platelet-to-lymphocyte ratio; SII, systemic immune-inflammation index; MHR, monocyte-to-HDL cholesterol ratio.

†Serum levels of Valine in quartiles: Q1, < 223.9 μmol/L; Q2, 223.9-255.4 μmol/L; Q3, 255.4-288.9 μmol/L; and Q4, ≥ 288.9 μmol/L.

*P＜0.05, significant difference.

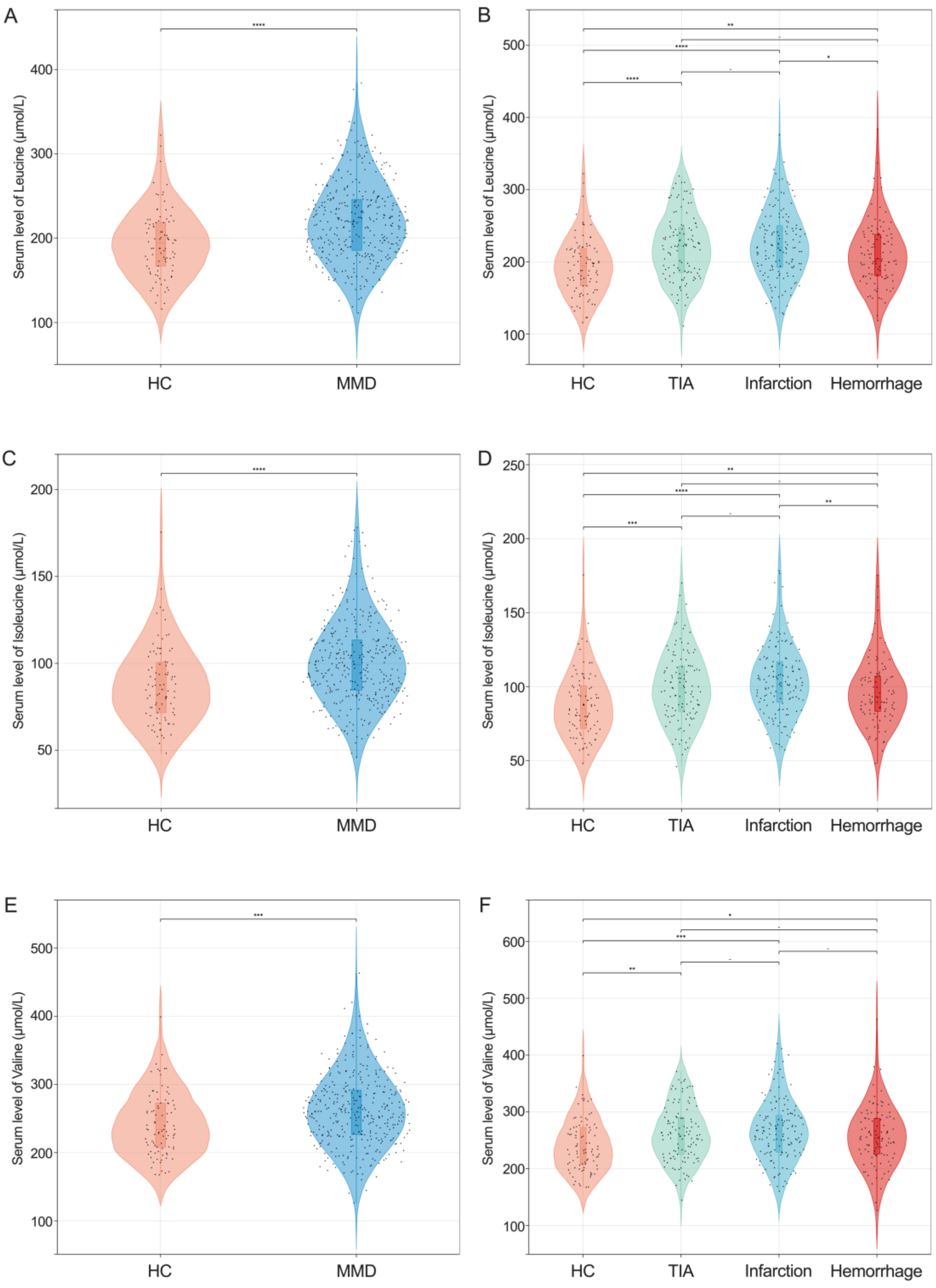

Figure S1. Quantitative analysis of serum individual BCAAs level between MMD and HCs.

HC, healthy control; MMD, moyamoya disease; TIA, transient ischemic attack.

*P < 0.05; **P < 0.01; ***P < 0.001; ****P < 0.0001.

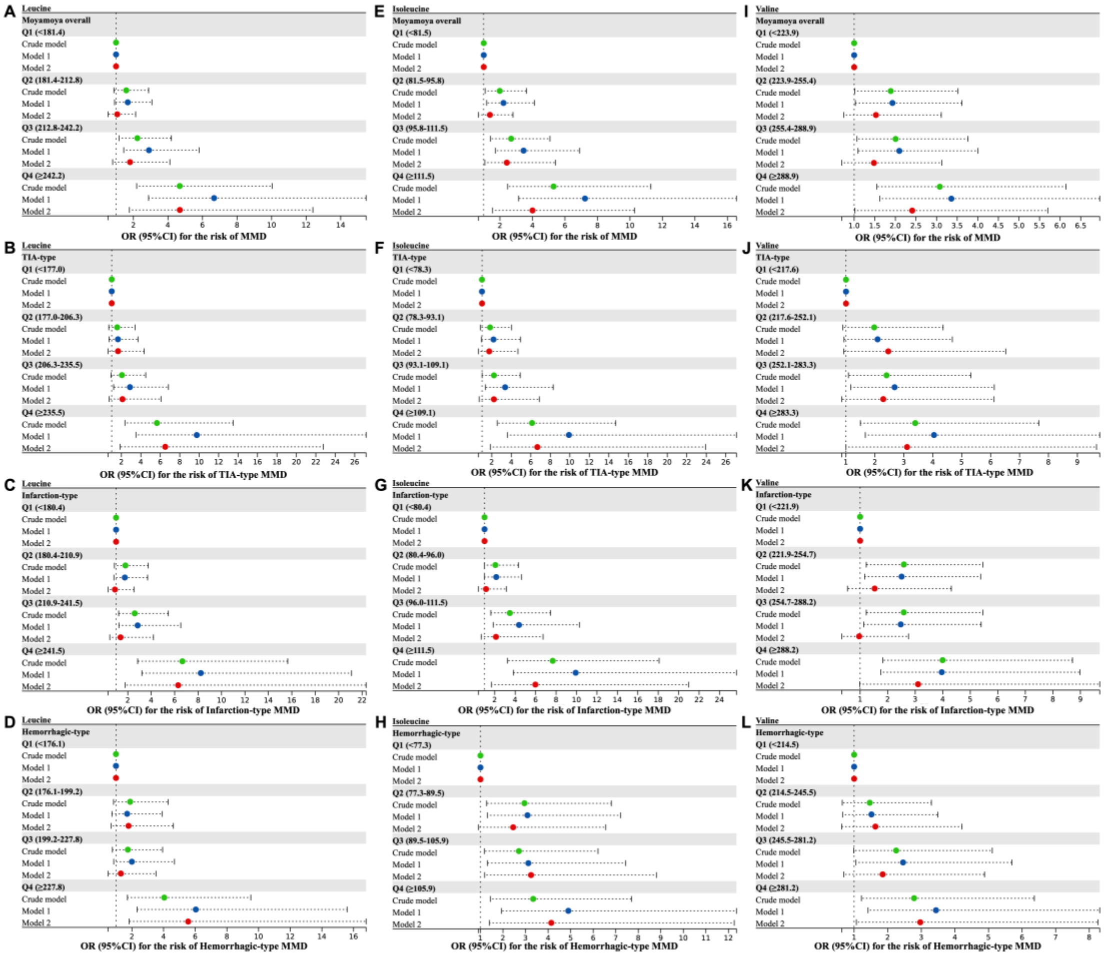

Figure S2. The association of circulating individual BCAAs level with the risk of MMD and clinical subtypes. A-D. Forest plots for the association of Leucine with MMDand subtypes. E-H. Forest plots for the association of Isoleucine with MMD and subtypes. I-L. Forest plots for the association of Valine with MMD and subtypes. Model 1, adjusted for age and sex. Model 2, further adjusted for BMI, WBC count, neutrophil count, glucose, TG, TC, HDL-C, LDL-C, APO-A_1, Hcy, NLR, SII, and MHR.

OR, odds ratio; CI, confidence interval; MMD, moyamoya disease; TIA, transient ischemic attack.

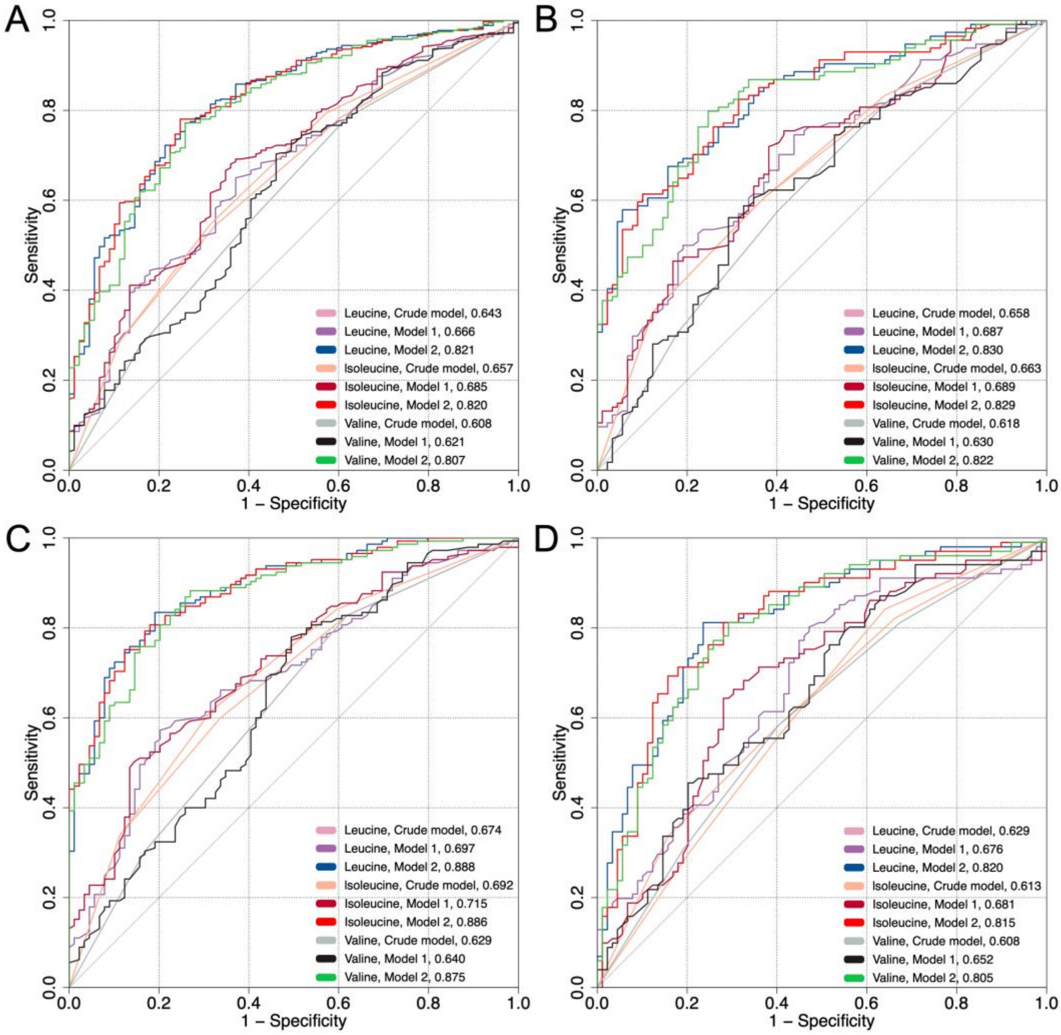

Figure S3. ROC curves with AUC of different models for the risk of MMD and subtypes. A. MMD overall. B. TIA-type MMD. C. Infarction-type MMD. D. Hemorrhagic-type MMD. Model 1, adjusted for age and sex. Model 2, further adjusted for BMI, WBC count, neutrophil count, glucose, TG, TC, HDL-C, LDL-C, APO-A_1, Hcy, NLR, SII, and MHR.
